# Association of three SNPs in *TOX3* and breast cancer risk: Evidence from 97275 cases and 128686 controls

**DOI:** 10.1038/srep12773

**Published:** 2015-08-04

**Authors:** Li Zhang, Xinghua Long

**Affiliations:** 1 Zhongnan Hospital of Wuhan University, Wuhan, 430071, China

## Abstract

The associations of SNPs in *TOX3* gene with breast cancer risk were investigated by some Genome-wide association studies and epidemiological studies, but the study results were contradictory. To derive a more precise estimate of the associations, we conducted a meta-analysis. ORs with 95% CI were used to assess the strength of association between *TOX3* polymorphisms and breast cancer risk in fixed or random effect model. A total of 37 publications with 97275 cases and 128686 controls were identified. We observed that the rs3803662 C > T, rs12443621 A > G and rs8051542 C > T were all correlated with increased risk of breast cancer. In the stratified analyses by ethnicity, significantly elevated risk was detected for all genetic models of the three SNPs in Caucasians. In Asian populations, there were significant associations of rs3803662 and rs8051542 with breast cancer risk. Whereas there was no evidence for statistical significant association between the three SNPs and breast cancer risk in Africans. Additionally, we observed different associations of rs3803662 with breast cancer risk based on different ER subtype and *BRCA1/BRCA2* mutation carriers. In conclusion, the meta-analysis suggested that three SNPs in *TOX3* were significantly associated with breast cancer risk in different populations.

Breast cancer is the most generally diagnosed cancer and the most common cause of cancer death for females all over the world, particularly in the economically developing countries^1^. It is well known that breast cancer is a heterogeneous disease, not only in the aspect of various pathogenesis, but also in diversified clinical manifestation and outcome. Meanwhile, breast carcinoma is multifactorial disease, from a certain perspective, along with the combination of polygenic inheritance factor and environmental factor. Accompany with technological advances, more studies related with the genomic variation were conducted, in order to improve diagnosis and treatment for breast cancer patients. Mutations in some high and moderate penetrate genes, such as *BRCA1*, *BRCA2* and *ATM*, were verified to be connected with the increased risk of breast cancer^2^,^3^. Nonetheless, these mutations constitute a part of the disease risk and it remains unclarified for the majority of genetic variations related with breast cancer susceptibility, particularly for low penetrate genes. It is noteworthy that genome-wide association studies (GWASs) about hundreds of single nucleotide polymorphisms (SNPs) provide strong evidences in elaborating the associations between low penetrate genes and breast cancer risk.

The *TOX3* gene, formerly known as trinucleotide repeat containing 9 (*TNRC9*), is located in the chromosome 16q 12 and has a tri-nucleotide repeat motive. The gene encoded a protein containing a putative high mobility group (HMG) box^4^, indicating that it might play a potential role in calcium dependent transcription as a transcription factor^5^. In the recent years, the associations between genetic variants in *TOX3* region and breast cancer susceptibility have been validated by GWASs and epidemiological studies in European, Asian and African American populations^6^,^7^,^8^,^9^,^10^,^11^,^12^,^13^. The SNP rs3803662 is located in 8 kb upstream of *TOX3*, and the rs12443621 and rs8051542 are both lied in an linkage disequilibrium (LD) block containing the 5′ end of *TOX3*^6^.

The *TOX3* rs3803662 was identified to exhibit association with breast cancer by GWASs^6^,^7^,^10^, with ascertainment of the association in Hispanic and non-Hispanic white women by Slattery *et al.*^11^. However, no significant association was found between rs3803662 and breast cancer risk in Asian and African ancestry^8^,^13^. Analogously, there was no evidence for the association between rs12443621 or rs8051542 and increased risk of breast cancer in Chinese women^14^,^15^,^16^. Whereas, Shan *et al.* reported that rs8051542 was significantly correlated with breast cancer risk in Tunisians^17^. Additionally, some studies found different relationships of three SNPs and breast cancer risk among different populations, which might result from different sample size or diverse allele frequencies and LD pattern among populations.

Meanwhile, the most recent meta-analysis related to the associations between the above-mentioned 3SNPs with breast cancer risk omitted some important studies^18^, and thus had limited statistical power to demonstrate the associations. Therefore, we performed an updated meta-analysis to aim to come up with the highest level of evidence for the associations between three SNPs in *TOX3* gene and breast cancer risk among diverse ancestry populations and distinct tumor subtypes stratified by estrogen receptor (ER) or *BRCA1/BRCA2.*

## Materials and Methods

### Literature search strategy

We carried out a comprehensive literature search from PubMed and EMBASE databases up to March 2015, using the following search terms “*TOX3*” or “*TNRC9*” and “polymorphism” or “genetic variant” or “rs3803662” or “rs12443621” or “rs8051542” and “breast cancer” or “breast carcinoma” or “breast tumor” . First, we retrieved all potentially relevant articles, whose abstracts contained information related to our research purpose. Second, the references from eligible studies were carefully checked for additional relevant literature. Finally, only the comprehensive or the most recent study was brought into this meta-analysis, in the case that the same study population was included in several different articles.

### Selection criteria

Eligible studies had to fulfill the following criteria: (1) case-control studies or cohort studies evaluating the association between *TOX3* polymorphism (rs3803662, rs12443621 or rs8051542) and breast cancer risk; (2) odds ratio (OR) and 95% confidence interval (CI) or genotype data of rs3803662, rs12443621 or rs8051542 in breast cancer patients and cancer-free female to calculate OR and 95% CI; (3) studies were confined to human female groups; (4) articles in English.

### Data extraction

A standard protocol was applied to extract data. For every eligible study, the following data were extracted: First author’s surname, year of publication, country of origin, population ethnicity, genotyping method, the genotype counts in cases and control (TT, CT and CC genotypes for *TOX3* rs3803662; GG, AG and AA genotypes for rs12443621; TT, CT and CC genotypes for rs8051542) and P-value for the HWE in control groups. Two investigators independently extracted the above relative data with any disagreement resolved by discussion. If no consensus wasn’t reached, another investigator joined in the discussion. And the final decision was made by the majority of the votes.

### Statistical methods

The strength of associations between *TOX3* polymorphisms and breast carcinoma risk were estimated by OR with corresponding 95% CI. For all studies, we assessed the association under five different genetic models for calculating OR. Those were homozygote codominant model, heterozygote codominant model, dominant model, recessive model and allele model. Hardy-Weinberg equilibrium (HWE) was assessed by using χ^2^ test to compare expected and actual genotype frequencies among controls of each study. Q-statistic was applied to investigate heterogeneity among studies. P-value greater than 0.1 for Q test suggested a lack of statistically significant heterogeneity, and the fixed-effect model (Mantel-Haenszel method)^19^ was used to calculate pooled ORs. Otherwise, heterogeneity was present and the random-effect model (DerSimonian-Laird method)^20^ was more appropriate. In addition, the *I*^*2*^-test was employed to accurately measure the degree of heterogeneity. Furthermore, the *I*^*2*^-value less than 25% was equivalent to mild heterogeneity, and values between 25% and 50% was equivalent to moderate heterogeneity, whereas values greater than 50% was equivalent to large heterogeneity among studies. Potential publication bias was estimated by symmetry of funnel plot of OR versus the standard error of log (OR) and the visual symmetrical plot indicated that there was no publication bias among studies. Sensitivity analyses were conducted to assess the robustness of the results by eliminating each study in turn to show whether the individual data set influenced the pooled OR. Stratified analyses were conducted in terms of ethnicity, estrogen receptor (ER) status, *BRCA1* and *BRCA2* mutation. All statistical tests in this meta-analysis were two-tailed and P-value ≤ 0.05 was considered statistically significant unless otherwise noted. All statistical analyses were performed with Review Manager 5.2 software recommended by Cochrane Collaboration and Comprehensive Meta Analysis V2 software.

## Result

### Study Characteristics

**Figure 1:**
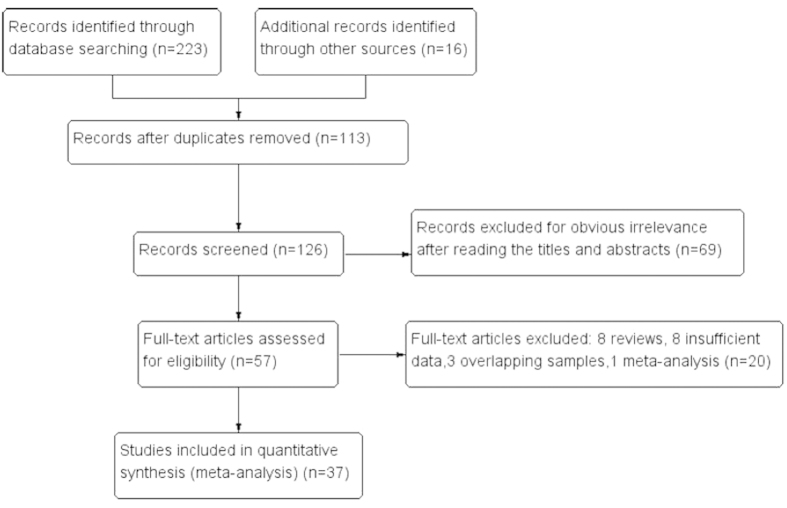
The flowchart of the study selection process.

Based on the above selection criteria, a total of 37 eligible studies were included in the pooled analyses, involving 97275 cases and 128686 controls for rs3803662 polymorphism^7^,^8^,^9^,^10^,^11^,^12^,^13^,^14^,^15^,^16^,^17^,^21^,^22^,^23^,^24^,^25^,^26^,^27^,^28^,^29^,^30^,^31^,^32^,^33^,^34^,^35^,^36^,^37^,^38^,^39^,^40^,^41^,^42^,^43^,^44^,^45^,^46^. For rs12443621, 14 studies^8^,^12^,^14^,^15^,^16^,^17^,^24^,^28^,^29^,^31^,^41^,^42^,^45^,^46^ involved a total of 17750 cases and 19488 controls. Moreover, there were 13 studies^8^,^12^,^15^,^16^,^17^,^28^,^29^,^31^,^39^,^41^,^42^,^45^,^46^ with 20965 cases and 21580 controls for rs8051542. Of particular note was that it’s smaller than 0.05 for the P-value of Hardy-Weinberg equilibrium in the controls of two studies, Campa *et al.* and Garcia-Closas *et al.*^37^,^38^, but we still included the two studies after sensitivity analyses were done. Additionally, in three included studies, genotype frequencies were shown separately according to different ethnic groups^7^,^31^,^39^. Therefore, the corresponding genotype counts in the study were separately considered for analyses. For rs3803662, five studies^11^,^15^,^16^,^26^,^38^ concerned with ER subtype of breast cancers and three studies^26^,^30^,^35^ related with BRCA1/2 mutation carriers were analysed as subgroups. The Fig. 1 expounded the study selection process. The Table 1 and 2 described the main features of these studies, especially for the genotype counts.

### Meta-analysis results

**Figure 2:**
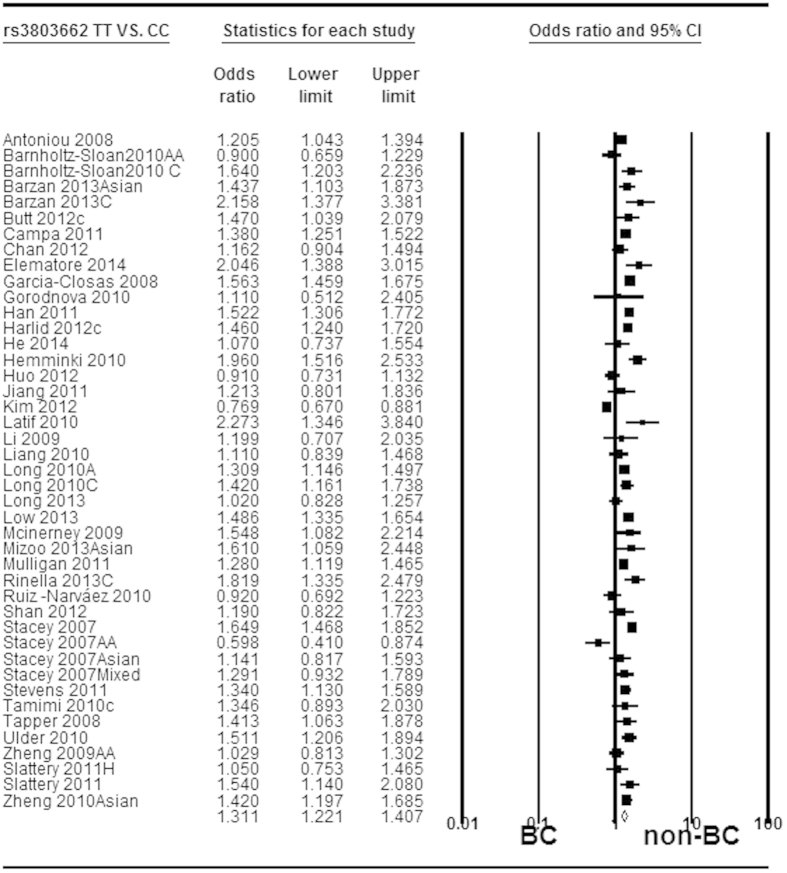
Forest plot of *TOX3* rs3803662 polymorphism and breast cancer risk. Random-effect model was used for the analysis (homozygote codominant model TT vs. CC). The squares and horizontal lines correspond to the specific OR and 95% CI for every study. The area of the squares reflects the study specific weight. The diamond stands for the pooled OR and 95% CI.

**Figure 3:**
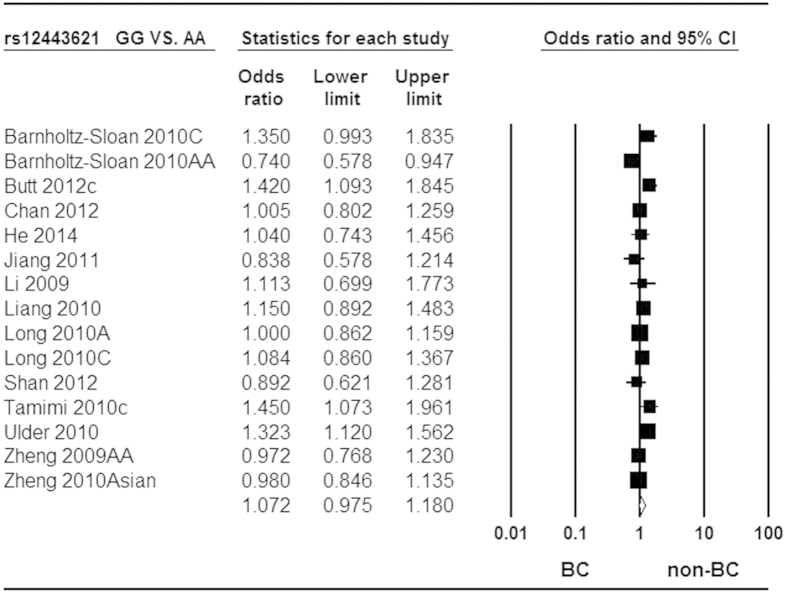
Forest plot of *TOX3* rs12443621 polymorphism and breast cancer risk. Random-effect model was used for the analysis (homozygote codominant model GG vs. AA).

**Figure 4:**
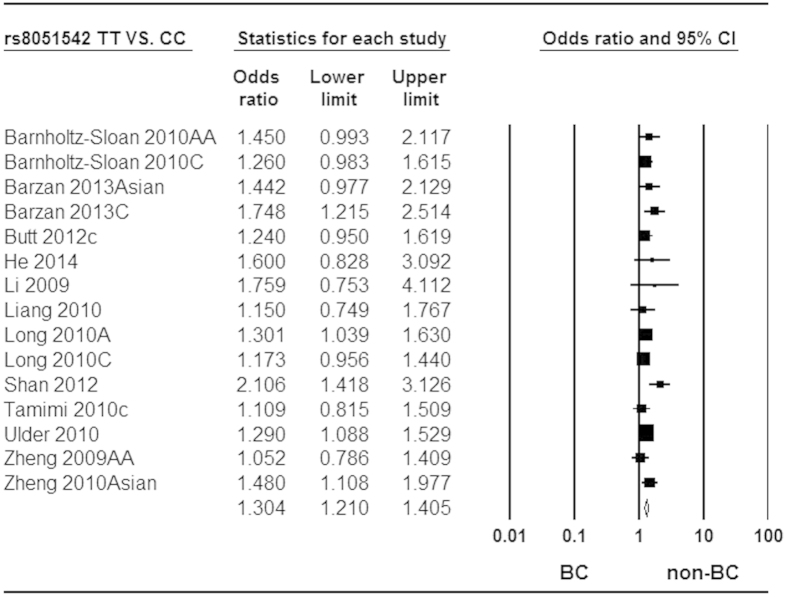
Forest plot of *TOX3* rs8051542 polymorphism and breast cancer risk. Fixed-effect model was used for the analysis (homozygote codominant model TT vs. CC).

The mixtures of adjusted and crude estimates were used to calculate pooled ORs. The available adjusted variables of included studies were listed in supplementary table 1. Owing to large heterogeneity among studies, we used random-effect model to calculate pooled ORs for the associations of rs3803662 and rs12443621 with breast cancer risk. In contrast, fix-effect model was applied to calculate pooled ORs for rs8051542. In aggregate, T-rs3803662 and T-rs8051542 were all statistically associated with increased risk of breast cancer in all genetic models. However, the association between G-rs12443621 and breast cancer risk was only observed in Caucasians under all genetic models. The pooled ORs and 95%CI for these associations in all genetic models were shown in detail in Table 3, 4, 5, respectively. Forest plots related to the association of rs3803662, rs12443621 and rs8051542 with breast cancer susceptibility in homozygote model were shown in Fig. 2, Fig. 3 and Fig. 4, respectively.

In the subgroup analysis by ethnicity, our results indicated statistically significant associations between the three SNPs and breast cancer susceptibility in Caucasians under all genetic models. Nevertheless, as for Asian populations, T-rs3803662 and T-rs8051542 were shown to be statistically significant correlated with increased risk of breast cancer in all genetic models. In addition, there was no evidence for the statistical significant associations between the three SNPs and increased risk of breast cancer in African population which were almost African-American in our study. The pool ORs and 95%CI for these stratified analyses were detailedly shown in Table 3, 4, 5 for all genetic modes.

**Figure 5:**
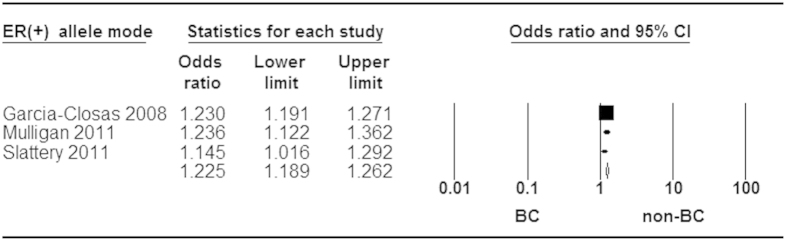
Forest plot of *TOX3* rs3803662 polymorphism and breast cancer risk stratified by ER (+). Fixed-effect model was used for the analysis (allele model T vs. C).

**Figure 6:**
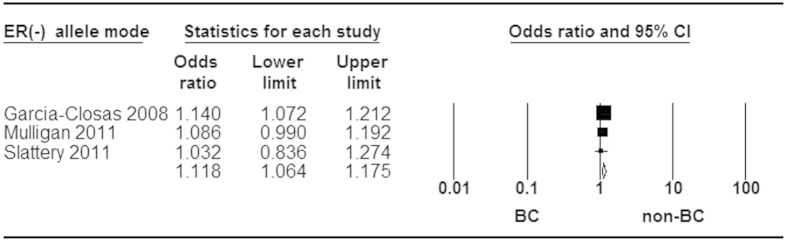
Forest plot of *TOX3* rs3803662 polymorphism and breast cancer risk stratified by ER (−). Fixed-effect model was used for the analysis (allele model T vs. C).

**Figure 7:**
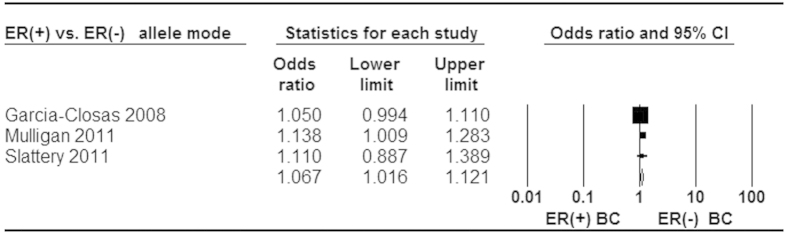
Forest plot of *TOX3* rs3803662 polymorphism and breast cancer risk in ER+ subtype compared with ER− tumors. Fixed-effect model was used for the analysis (allele model T vs. C).

**Figure 8:**
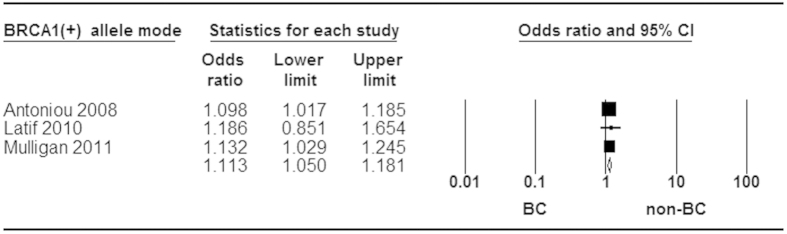
Forest plot of *TOX3* rs3803662 polymorphism and breast cancer risk stratified by BRCA1 mutation. Fixed-effect model was used for the analysis (allele contrast model T vs. C).

**Figure 9:**
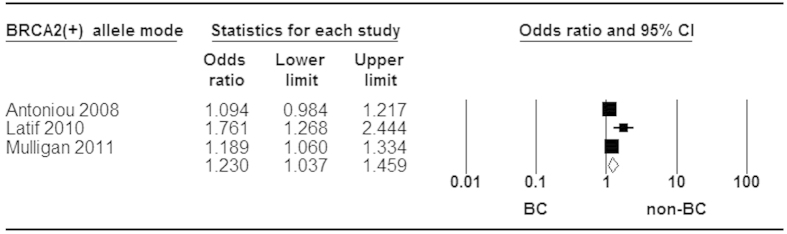
Forest plot of *TOX3* rs3803662 polymorphism and breast cancer risk stratified by BRCA2 mutation. Fix -effect model was used for the analysis (allele contrast model T vs. C).

When stratified by ER status for rs3803662, statistically significant increased risk was found in ER^+^ and ER^−^ tumor (Fig. 5 and Fig. 6). Moreover, a stronger association was identified in ER^+^ than ER^−^ subtype for breast cancer risk (Fig. 7). Additionally, our analysis demonstrated that there were significant relationships between elevated risk of breast cancer and *BRCA1/2* mutation carriers for rs3803662 (Fig. 8 and Fig. 9). And the details about ORs and 95% CI under all genetic models were shown in Table 3.

### Sensitivity analyses and publication bias

Sensitivity analyses were conducted to assess the robustness of the results by eliminating each study in turn and all the results were not essentially altered, suggesting that the results of our meta-analysis were statistically stable. Publication bias of the eligible literature was evaluated by funnel plots and the shapes of funnel plots for literature about association between three SNPs and breast cancer risk were mostly symmetrical, indicating that no publication bias was detected.

## Discussion

The *TOX3* gene encoded a protein with an HMG box that is considered to be implicated in modification of DNA and chromatin structure^47^. Moreover, increased expression of *TOX3* was relevant to bone metastasis in breast cancer patients^48^. Whereas, precise biological function of *TOX3* is undetermined. Some GWASs and epidemiological studies have identified the associations of *TOX3* polymorphisms with breast cancer susceptibility. However, study results were not consistent. Hence, in order to resolve the conflict, we performed this meta-analysis of the associations between the *TOX3* rs3803662, rs12443621 and rs8051542 polymorphism and breast cancer risk.

The three SNPs locate in the 5’ end of *TOX3* gene and a hypothetical gene LOC643714 on 16q12, and the region is contained in a 133kb linkage disequilibrium (LD) block^12^. Based on the International HapMap database, different LD patterns were observed between Asian and European ancestry. SNP rs3803662 was in moderate LD with rs12443621, with a Pearson’s correlation coefficient (r^2^) of 0.29 in the HapMap CEU population for European ancestry, but very weak LD was found between these two SNPs (r^2^ = 0.06) in Chinese^8^. Similarly, there was very weak association (r^2^ = 0.08) between rs3803662 and rs8051542 located 52 kb apart from each other in Chinese women^45^. However, the two SNPs showed moderate association (r^2^ = 0.15) with each other in European populations^8^. The substantial differences in genetic architecture among races, such as allele frequencies and LD structures, may partly account for our results which confirmed different association of the three SNPs with breast cancer risk in Caucasians, Asians and African-Americans. Rs3803662-T allele, rs12443621-G allele and rs8051542-T allele were statistically significantly associated with increased risk of breast cancer in Caucasians. Meanwhile, T-rs3803662 and T-rs8051542 were identified as risk factors of breast cancer in Asian populations. However, there was no evidence to prove that the three SNPs in African-Americans and G-rs12443621 in Asians were implicated in the breast tumor susceptibility, which was in line with the previous studies^41^,^45^. It’s worth mentioning that our study showed that T-rs3803662 and G-rs12443621 were protective factors in African-Americans in spite of no statistical significance.

In general, our study proved that the T-rs3803662 and T-rs8051542 in *TOX3* were correlated with elevated breast cancer risk in all genetic models. It is notable that a previous meta-analysis directed by Chen *et al.*^18^ has showed that rs3803662 polymorphism was significantly associated with breast cancer risk, but no significant associations were observed for the rs12443621 and rs8051542. In addition, it only included eight case-control studies without stratified analyses. Compared with the previous meta-analysis, our study had more powerful and detailed analyses to prove our results. First and most obviously, more eligible literature and larger sample size were included. Second, the associations between breast cancer risk and rs3803662 polymorphism were considered with respect to ER status and BRCA1/2 mutation carriers. Third, stratified analyses were performed based on Caucasians, Asians and Africans, which was in favor of a more comprehensive understanding the associations in diverse populations. Finally and most importantly, we used mixture of adjusted and crude ORs rather than unadjusted estimates to calculate the pooled ORs. Meanwhile, the original genotype counts of eligible studies were also used to calculate the crude ORs. Supplementary table 2 showed the pooled ORs of the associations between the 3SNPs and breast cancer risk by using crude estimates. And there was no significant difference among the two results of pooled ORs based on different estimates, except for rs12443621. The crude ORs were incorporated to result in marginally association of rs12443621 with breast cancer risk under homozygote, dominant and allele genetic mode, but no association was found by using mixture ORs. That was probably because that adjusted estimates could yield more accurate results. Nevertheless, the two ways both demonstrated the relationship between rs12443621 and elevated risk of breast cancer in Caucasians.

To date, more attention has been paid to the heterogeneity of associations between common genetic variants and breast cancer subtypes. The two large-scale studies^37^,^38^ and our result identified that rs3803662 polymorphism was associated with both ER^+^ and ER^−^ subtype of breast cancer, in spite of the slightly weaker association for ER^−^ breast cancer. Additionally, our study demonstrated that T-rs3803662 was statistically significant associated with increased risk in ER^+^ breast cancer compared with ER^−^ subtype, which was accordance with the researches done by Stacey *et al.* and Broeks *et al.*^7^,^49^. Intriguingly, T-rs8051542 allele and rs12443621 AG/GG genotypes, rather than rs3803662, were significantly associated with elevated risk of ER^+^ breast cancer in Chinese women^8^,^16^. By contrast, the significant associations of rs8051542 and rs12443621 were observed with luminal A (ER/PR^+^, Her2^−^) and Her2^+^/ER^−^ breast cancer only among whites, respectively^50^. Furthermore, the association was strongly confirmed between rs3803662 and triple-negative tumors^25^,^49^. Taken together, these studies indicated that there were somehow connections between the three SNPs in *TOX3* gene and pathological subtype of breast tumor among different populations. And in another aspect, these studies provided further support for the hypothesis that different subtypes stem from diverse etiological pathways. Additionally, rs3803662 SNP in *BRCA1* and *BRCA2* mutation carriers was significantly associated with the increased risk of breast cancer in our analysis, which was in consistent with previous studies^26^,^35^. While Latif *et al.* confirmed that the genetic variant was only associated with breast tumor in *BRCA2* mutation carriers^30^. Therefore, it’s necessary to further elucidate the relevance of rs3803662 to breast cancer risk with *BRAC1* and *BRCA2* mutation.

Despite the advantage of large sample size and stratified analyses, the meta-analysis had several limitations that should be taken into account. First, there was extreme heterogeneity for the outcomes of the association between rs3803662 polymorphism and breast cancer risk. Although we reduced the degree of heterogeneity by stratified analyses based on ethnicity, other sources of heterogeneity were not verified, such as different genotyping methods or tumor types. Second, the sample size of African populations (5462 cases and 7155 controls) was relatively small. Therefore there was insufficient statistical power to demonstrate the associations between the 3SNPs and breast cancer risk in Africans. Third, the criterion of control groups was not uniformly defined. The design of eligible studies was based on population or/and hospital patients, thus there were potential risks of breast cancer in control groups. Fourth, the mixtures of crude and adjusted publish estimates, rather than incorporation of adjusted ORs, were used in the meta-analysis. Because of the lack of some individual data, we were unable to adjust effect size with possible confounders related with lifestyle risk factors, such as age, obesity, smoking, alcohol consumption and menopausal status. Furthermore, we were unable to examine the interaction between genetic variables and environment. In recent years, some studies for gene-environment interactions showed that relative risks of breast cancer correlated with low-penetrance susceptibility variants (including rs3803662) didn’t vary significantly with established environmental risk factors, such as reproductive history, menopausal status and body mass index^51^,^52^,^53^. Nevertheless, more and more researches have elaborated combined effect of low-penetrance susceptibility loci with breast cancer risk. And the obviously elevated risk stemming from combining many low-penetrant risk alleles supports the polygenic inheritance model of breast cancer^44^. Finally, owing to merely include English articles, there might be language bias on some level. Additionally, positive reports are tended to be published, which might make certain bias.

In conclusion, this meta-analysis indicated that there were different associations between the 3SNPs in *TOX3* gene and breast cancer risk in different ethnic groups or subtype tumor. The 3SNPs were associated with the increased risk of breast cancer in Caucasians, while weren’t correlated in Africans. Additionally, rs3803662 and rs8051542 were risk factors for breast cancer in Asians. Furthermore, there were stronger associations between rs3803662 polymorphism and breast cancer risk in ER^+^ subtype than ER^−^ tumors. Increased risk of breast cancer associated with rs3803662 was confirmed in *BRCA1/BRCA2* mutation carriers. However, studies with larger sample size, which use uniform genotyping methods and criterion of control groups, have sufficiently corresponding individual data and consider the interactions of gene-gene and gene-environment will be needed to verify our results for *TOX3* rs3803662, rs12443621 and rs8051542 as predisposition markers to breast cancer in clinical application.

## Additional Information

**How to cite this article**: Zhang, L. and Long, X. Association of three SNPs in *TOX3* and breast cancer risk: Evidence from 97275 cases and 128686 controls. *Sci. Rep.* **5**, 12773; doi: 10.1038/srep12773 (2015).

## Supplementary Material

supplementary table

## Figures and Tables

**Table 1:** Characteristics of studies for the association of *TOX3* rs3803662 with breast cancer risk included in the meta-analysis.

Author	Year	Country	Ethnicity	Genotyping method	Case			Control			
					TT	CT	CC	TT	CT	CC	P_HWE_
Stacey^7^	2007	Iceland	Caucasian	Illumina	469	1985	2100	1272	6913	9392	0.999
Stacey^7^	2007	Iceland	AA	Illumina	95	211	116	130	222	95	0.990
Stacey^7^	2007	Iceland	Asian	Illumina	155	275	122	147	278	132	0.980
Stacey^7^	2007	Iceland	Mixed	Illumina	104	275	183	114	340	259	0.891
He^8^	2014	China	Asian	Sequenom MassARRAY	271	280	72	270	278	72	0.973
Elematore^9^	2014	Chile	Mixed	TaqMan	62	185	100	100	371	330	0.786
Low^10^	2013	Japan	Asian	Illumina OmniExpress BeadChip	1801	2705	1016	1496	2739	1254	0.996
Slattery^11^	2011	USA	Caucasian	TaqMan	204	755	778	202	862	978	0.550
Udler^12^	2010	UK	Caucasian	NA	194	942	1041	157	829	1273	0.167
Ruiz -Narváez^13^	2010	USA	AA	Sequenom MassArray iPLEX	188	376	189	214	412	199	0.980
Jiang^14^	2011	China	Asian	SNaPshot	233	212	48	232	224	54	0.995
Li^15^	2009	China	Asian	PCR-LDR	118	141	32	123	128	40	0.470
Liang^16^	2010	China	Asian	SNP stream highthroughput 12-plex	486	413	126	455	464	127	0.603
Shan^17^	2012	Qatar	Caucasian	TaqMan	147	293	200	78	165	126	0.083
Long^21^	2013	USA	AA	TaqMan/Sequenom	328	613	287	563	1027	469	0.988
Kim^22^	2012	Korea	Asian	Affymetrix/TaqMan	499	1939	1887	577	1967	1677	0.996
Huo^23^	2012	USA	African	Illumina GoldenGate	393	754	362	368	691	324	0.991
Chan^24^	2012	Singapore	Asian	TaqMan	541	499	134	629	656	181	0.622
Stevens^25^	2011	USA	Caucasian	iPLEX/Illumina	268	1252	1460	363	1960	2650	0.982
Mulligan^26^	2011	Canada	Mixed	TaqMan	585	2652	3109	426	2197	2899	0.730
Han^27^	2011	Korea	Asian	RT-PCR	1481	1435	369	1361	1617	516	0.317
Long^28^	2010	USA	Asian	Affymetrix/Sequenom MassARRAY	2934	2761	650	1603	1727	465	0.996
Long^29^	2010	USA	Caucasian	TaqMan	258	1172	1330	190	1028	1391	0.997
Latif^30^	2010	UK	Caucasian	TaqMan	84	395	422	19	137	217	0.660
Barnholtz-Sloan^31^	2010	USA	AA	Illumina GoldenGate	196	378	166	182	333	142	0.654
Barnholtz-Sloan^31^	2010	USA	Caucasian	Illumina GoldenGate	133	512	585	89	440	589	0.591
Gorodnova^32^	2010	Russia	Caucasian	RT-PCR	16	50	74	15	82	77	0.294
Hemminki^33^	2010	Germany	Caucasian	MALDITOF mass spectrometry	154	626	635	124	704	1002	0.982
Mcinerney^34^	2009	Ireland	Caucasian	KASPar SNP genotyping	82	382	486	58	396	532	0.161
Antoniou^35^	2008	UK	Caucasian	TaqMan/MALOI-TOF/iPLEX	497	2173	2422	382	1831	2244	0.756
Tapper^36^	2008	UK	Caucasian	iPLEX Service	76	371	452	196	1137	1647	0.990
Campa^37^	2011	Germany	Mixed	TaqMan	1071	3528	3706	1150	4724	5721	0.001
Garcia-Closas^38^	2008	USA	Mixed	TaqMan/MALDITOF	1848	7132	7759	2026	9705	13295	<0.0001
Barzan^39^	2013	Germany	Asian	Sequenom MassARRAY	482	413	89	961	990	255	0.999
Barzan^39^	2013	Germany	Caucasian	Sequenom MassARRAY	36	140	135	65	369	526	0.979
Rinella^40^	2013	USA	Caucasian	Affymetrix/KASPar	131	335	214	106	366	315	0.985
Zheng^41^	2009	USA	AA	Sequenom MassARRAY	222	404	184	482	891	411	0.984
Butt^42^	2012	Sweden	Caucasian	Sequenom MassARRAY	64	278	353	95	512	780	0.380
Mizoo^43^	2013	Japan	Asian	TaqMan	160	230	74	142	227	91	0.987
Harlid^44^	2012	Sweden	Caucasian	MALDITOF mass spectrometry	330	1420	1794	352	1898	2768	0.280
Zheng^45^	2010	USA	Asian	Affymetrix	1401	1325	313	1286	1410	386	0.987
Tamimi^46^	2010	USA	Caucasian	Sequenom iPLEX/TaqMan	54	300	333	50	273	415	0.576

AA: African Americans; NA: not available; HWE: Hardy-Weinberg equilibrium; PCR-LDR : Polymerase chain reaction–ligation detection reaction.

**Table 2:** Genotypes and P_WHE_ for *TOX3* rs12443621 and rs8051542 polymorphisms included in the study.

Author	rs12443621 Genotypes						P_HWE_	rs8051542 Genotypes						P_HWE_
	Cases			Controls				Cases			Controls			
	GG	AG	AA	GG	AG	AA		TT	CT	CC	TT	CT	CC	
He^8^ 2014	110	304	209	115	304	201	0.809	25	199	399	18	175	427	0.989
Udler^12^ 2010	527	1111	546	497	1099	681	0.176	455	1089	611	425	1067	736	0.274
Jiang^14^ 2011	170	239	84	162	251	97	0.990	—	—	—	—	—	—	—
Li^15^ 2009	106	138	54	97	141	55	0.766	15	82	198	9	90	209	0.854
Liang^16^ 2010	347	507	186	338	519	204	0.850	48	314	670	47	309	708	0.078
Shan^17^ 2012	190	301	147	98	180	85	0.894	138	289	208	46	176	146	0.529
Chan^24^2012	404	573	198	532	669	262	0.419	—	—	—	—	—	—	—
Long^28^ 2010	546	1448	960	554	1469	974	0.998	246	1971	3941	118	1080	2460	0.968
Long^29^ 2010	286	573	286	274	571	297	0.989	336	788	463	279	709	451	0.991
Barnholtz-Sloan^31^ 2010	164	370	208	165	329	164	0.999	87	342	313	59	304	295	0.121
Barnholtz-Sloan^31^ 2010	337	580	313	242	574	302	0.319	257	587	386	201	559	358	0.501
Barzan^39^ 2013	—	—	—	—	—	—	—	43	327	614	72	651	1483	0.957
Barzan^39^ 2013	—	—	—	—	—	—	—	81	155	75	186	473	301	0.994
Zheng^41^ 2009	189	405	216	423	891	470	0.986	80	349	381	170	762	852	0.984
Butt^42^ 2012	165	338	195	275	657	451	0.203	149	338	192	272	637	443	0.119
Zheng^45^ 2010	1001	1486	552	1008	1509	565	0.995	118	961	1960	96	898	2088	0.963
Tamimi^46^ 2010	151	337	193	130	366	241	0.659	132	359	194	135	380	220	0.193

P_HWE_: P value of Hardy-Weinberg equilibrium for control groups.

**Table 3:** Stratified analysis of *TOX3* rs3803662 polymorphism on breast cancer.

Variables	N	TT versus CC		CT versus CC		TT + CT versus CC		TT versus CT + CC		T versus C	
		OR (95% CI)	P_H_	OR (95% CI)	P_H_	OR (95% CI)	P_H_	OR (95% CI)	P_H_	OR (95% CI)	P_H_
Total	43	1.311 (1.221–1.407)	<0.001	1.151 (1.103–1.201)	<0.001	1.160 (1.088–1.237)	<0.001	1.200 (1.140–1.263)	<0.001	1.145 (1.106–1.186)	<0.001
Ethnicity											
Asian	13	1.245 (1.076–1.440)	<0.001	1.121 (1.013–1.241)	<0.001	1.162 (1.030–1.311)	<0.001	1.133 (1.049–1.223)	<0.001	1.112 (1.034–1.195)	<0.001
Caucasian	19	1.483 (1.371–1.604)	0.029	1.212 (1.153–1.275)	0.005	1.259 (1.194–1.327)	<0.001	1.347 (1.280–1.418)	0.332	1.220 (1.171–1.271)	<0.001
African	6	0.929 (0.837–1.032)	0.240	0.961 (0.841–1.099)	0.044	0.989 (0.877–1.116)	0.048	0.952 (0.878–1.032)	0.52	0.962 (0.914–1.012)	0.296
Mixed	5	1.440 (1.288–1.611)	0.017	1.200 (1.109–1.298)	0.004	1.084 (0.927–1.268)	<0.001	1.358 (1.294–1.425)	0.265	1.202 (1.126–1.282)	<0.001
ER (+)	4	1.493 (1.391–1.603)	0.696	1.241 (1.188–1.297)	0.468	1.312 (1.262–1.364)	0.284	1.410 (1.321–1.505)	0.594	1.225 (1.189–1.262)	0.524
ER (−)	4	1.104 (0.885–1.376)	0.073	1.134 (1.066–1.206)	0.491	1.135 (1.008–1.278)	0.079	1.282 (1.163–1.414)	0.327	1.118 (1.064–1.175)	0.519
ER (+) vs. ER (−)	5	1.382 (0.998–1.915)	0.016	1.073 (1.002–1.149)	0.582	1.086 (1.019–1.157)	0.668	1.093 (0.986–1.212)	0.883	1.067 (1.016–1.121)	0.468
BRCA1	3	1.249 (1.087–1.436)	0.811	1.107 (1.022–1.198)	0.933	1.130 (1.048–1.219)	0.888	1.194 (1.044–1.365)	0.833	1.113 (1.050–1.181)	0.822
BRCA2	3	1.102 (0.921–1.319)	0.911	1.276 (1.018–1.599)	0.030	1.310 (1.039–1.650)	0.017	1.207 (1.018–1.432)	0.458	1.230 (1.037–1.459)	0.023

N: Numbers of data sets; P_H_: P-value of Q-test for heterogeneity test; P_H_ < 0.1 indicates that there is heterogeneity and random-effect model is used to calculate pooled OR_s_ and 95% CI. Otherwise, fixed-effect model is used.

**Table 4:** Stratified analysis of *TOX3* rs12443621 polymorphism on breast cancer.

Variables	N	GG versus AA		AG versus AA		GG + AG versus AA		GG versus AG + AA		G versus A	
		OR (95% CI)	P_H_	OR (95% CI)	P_H_	OR (95% CI)	P_H_	OR (95% CI)	P_H_	OR (95% CI)	P_H_
Total	15	1.072 (0.975–1.180)	0.005	1.033 (0.958–1.113)	0.020	1.049 (0.983–1.121)	0.047	1.061 (0.996–1.131)	0.069	1.033 (0.985–1.083)	0.003
Ethnicity											
Asian	7	1.006 (0.927–1.092)	0.885	1.002 (0.936–1.073)	0.663	1.006 (0.941–1.075)	0.696	1.007 (0.945–1.072)	0.802	1.000 (0.956–1.046)	0.843
Caucasian	6	1.264 (1.143–1.398)	0.201	1.156 (1.062–1.258)	0.281	1.163 (1.078–1.256)	0.147	1.181 (1.089–1.280)	0.273	1.125 (1.070–1.183)	0.190
African	2	0.854 (0.720–1.012)	0.117	0.863 (0.658–1.132)	0.062	0.931 (0.803–1.079)	0.355	0.926 (0.794–1.080)	0.371	0.928 (0.853–1.009)	0.145

N: Numbers of data sets; P_H_: P-value of Q-test for heterogeneity test; P_H_ <0.1 indicates that there is heterogeneity and random-effect model is used to calculate pooled OR_s_ and 95% CI. Otherwise, fixed-effect model is used.

**Table 5:** Stratified analysis of *TOX3* rs8051542 polymorphism on breast cancer.

Variables	N	TT versus CC		CT versus CC		TT + CT versus CC		TT versus CT + CC		T versus C	
		OR (95% CI)	P_H_	OR (95% CI)	P_H_	OR (95% CI)	P_H_	OR (95% CI)	P_H_	OR (95% CI)	P_H_
Total	15	1.304 (1.210–1.405)	0.378	1.125 (1.076–1.175)	0.647	1.159 (1.112–1.208)	0.689	1.198 (1.121–1.280)	0.420	1.135 (1.099–1.171)	0.388
Ethnicity											
Asian	6	1.370 (1.185–1.584)	0.886	1.141 (1.075–1.211)	0.812	1.164 (1.101–1.231)	0.917	1.200 (1.041–1.383)	0.708	1.148 (1.093–1.206)	0.786
Caucasian	7	1.29 (1.181–1.425)	0.107	1.128 (1.046–1.215)	0.281	1.199 (1.116–1.288)	0.557	1.226 (1.082–1.389)	0.079	1.138 (1.086–1.191)	0.137
African	2	1.186 (0.941–1.494)	0.189	1.030 (0.896–1.183)	0.917	1.030 (0.913–1.161)	0.996	1.145 (0.970–1.351)	0.407	1.066 (0.964–1.179)	0.318

N: Numbers of data sets; P_H_: P-value of Q-test for heterogeneity test; P_H_ <0.1 indicates that there is heterogeneity and random-effect model is used to calculate pooled OR_s_ and 95% CI. Otherwise, fixed-effect model is used.
