# Supplementary material for: Association of three SNPs in TOX3 and breast cancer risk: Evidence from 97275 cases and 128686 controls
Source: Sci Rep. 2015 Aug 4;5:12773. doi: 10.1038/srep12773 (PMC4523945; doi:10.1038/srep12773)
Supplement: supplementary table [file srep12773-s1.pdf]

Association of three SNPs in *TOX3* and breast cancer risk: Evidence from 97275 cases and 128686 controls

Li Zhang Xinghua Long*

Zhongnan Hospital of Wuhan University, Wuhan, 430071, China,

*Correspondence to xlong888@yahoo.com

Supplementary table 1. The available adjusted variables of included studies in the meta-analysis.

Study	Adjusted variables
He[8] 2014	Age
Slattery[11] 2011	Age, center and genetic admixture among Hispanic women
Ruiz -Narváez [13] 2010	Age, geographical region of residence and place of birth and European admixture
Jiang[14] 2011	Age and BMI
Liang[16] 2010	Age, age at menarche, menopausal status and family history of cancer
Long[21] 2013	Age, study site and the first ten principal components
Huo[23] 2012	Study site and African ancestry
Barnholtz-Sloan [31] 2010	Age, European ancestry and offset term
Campa[37] 2011	Age and subcohort
Butt[42] 2012	Age, year of inclusion in study, socioeconomic status and exposure to HRT
Mizoo[43] 2013	Age, BMI, smoking, meat ,mushroom, coffee, green tea, green and vegetable intake, leisure time excise and education
Harlid[44] 2012	Age
Zheng[45] 2010	Age, education level and recruitment phases in the SBCS (Ⅰ and Ⅱ)

Supplementary table 2. The pooled ORs of the associations between the 3SNPs and breast cancer risk by using crude estimates

SNP	Variables	Homozygote mode	Heterozygote mode	Dominant mode	Recessive mode	Allele mode
		OR(95% CI)	OR(95% CI)	OR(95% CI)	OR(95% CI)	OR(95% CI)
Rs3803662	Total	1.300(1.211-1.396)	1.152(1.106-1.199)	1.159(1.087-1.235)	1.200(1.140-1.263)	1.144(1.104-1.184)
	Asian	1.217(1.054-1.405)	1.099(0.999-1.209)	1.157(1.025-1.305)	1.133(1.049-1.223)	1.104(1.028-1.185)
	Caucasian	1.477(1.368-1.594)	1.214(1.154-1.277)	1.259(1.194-1.327)	1.347(1.280-1.418)	1.219(1.171-1.270)
	African	0.928(0.839-1.027)	0.964(0.881-1.054)	0.951(0.874-1.035)	0.952(0.878-1.032)	0.963(0.916-1.013)
	Mixed	1.457(1.315-1.614)	1.202(1.115-1.296)	1.084(0.927-1.268)	1.358(1.294-1.425)	1.210(1.141-1.282)
Rs12443621	Total	1.098(1.008-1.195)	1.050(0.999-1.105)	1.065(1.015-1.117)	1.061(0.996-1.131)	1.046(1.002-1.093)
	Asian	1.027(0.946-1.114)	1.023(0.953-1.099)	1.022(0.956-1.093)	1.007(0.945-1.072)	1.011(0.971-1.052)
	Caucasian	1.267(1.150-1.395)	1.117(1.030-1.211)	1.163(1.078-1.256)	1.181(1.089-1.280)	1.127(1.074-1.183)
	African	0.895(0.744-1.076)	0.949(0.811-1.109)	0.931(0.803-1.079)	0.926(0.794-1.080)	0.946(0.863-1.037)
Rs8051542	Total	1.281(1.190-1.379)	1.129(1.081-1.179)	1.156(1.109-1.204)	1.198(1.121-1.280)	1.132(1.097-1.168)
	Asian	1.313(1.139-1.514)	1.141(1.077-1.209)	1.159(1.096-1.225)	1.259(1.093-1.451)	1.144(1.091-1.200)
	Caucasian	1.313(1.151-1.497)	1.138(1.056-1.225)	1.179(1.100-1.265)	1.209(1.079-1.355)	1.135(1.084-1.188)
	African	1.172(0.933-1.473)	1.038(0.905-1.191)	1.061(0.931-1.209)	1.152(0.926-1.432)	1.065(0.965-1.175)
